# Resistant depressive disorder: electroconvulsive therapy versus esketamine

**DOI:** 10.1192/j.eurpsy.2025.1344

**Published:** 2025-08-26

**Authors:** M. P. Pando Fernández, P. Martínez Gimeno, M. A. Andreo Vidal, M. Calvo Valcárcel, M. D. L. Á. Guillén Soto, O. Martin Santiago, G. Medina Ojeda, A. Rodríguez Campos, L. Rodríguez Andrés, N. Navarro Barriga, B. Rodríguez Rodríguez, M. Fernández Lozano, M. J. Mateos Sexmero, A. Aparicio Parra, L. Rojas Vázquez, G. Lorenzo Chapatte, M. Ríos Vaquero, A. Monllor Lazarraga, L. Sobrino Conde, L. del Canto Martínez, F. J. González Zapatero, M. E. Espinosa Muth

**Affiliations:** 1 Psiquiatría; 2 HCUV, Valladolid, Spain

## Abstract

**Introduction:**

Electroconvulsive therapy (ECT) has long been regarded as highly effective in the treatment of Treatment-Resistant Depression (TRD), with remission rates ranging from 50% to 70%. In recent years, esketamine, an N-methyl-D-aspartate (NMDA) receptor antagonist, has emerged as a novel treatment option for TRD due to its rapid and potent antidepressant effects (1).

**Objectives:**

This case aims to reflect on the comparative use of esketamine versus ECT in the management of TRD, considering clinical outcomes, side effects, and patient response.

**Methods:**

A literature review was conducted using PubMed to gather evidence on the efficacy of esketamine and ECT for TRD. We also present a case study of a 53-year-old female patient with TRD to illustrate the clinical application and response to esketamine.

**Results:**

The patient, a 53-year-old divorced woman with a 31-year-old son, has a history of three psychiatric admissions in 2021 due to major depressive episodes. She was previously treated at a day hospital and continues follow-up care in outpatient settings. Her current psychopharmacological regimen includes Venlafaxine 300 mg and Mirtazapine 30 mg, with Aripiprazole recently discontinued.

The patient presented to the emergency department with a marked deterioration in mood, neglect of daily activities, and increased social isolation. Psychiatric evaluation revealed a depressed appearance, slowed speech, bradypsychia, and passive thoughts of death, though without suicidal ideation. Her biological rhythms were disrupted, and she experienced unquantified weight loss, despite good family support.

The patient received her first intranasal dose of esketamine (56 mg) without complications, followed by six additional doses twice weekly. Mild side effects included nausea and occasional depersonalization and derealization. Over time, the patient showed progressive improvement in both affective symptoms and daily functioning.

**Conclusions:**

This case highlights the rapid antidepressant effects of esketamine in TRD patients, aligning with current literature. Esketamine is noted for its quick action and efficacy compared to second-generation antipsychotics (2, 3). Despite these benefits, ECT remains a more cost-effective option with higher remission rates for TRD, even though it is slightly less expensive than esketamine (1). The patient experienced significant clinical improvement with esketamine, which was well-tolerated with minimal side effects. However, the long-term efficacy and cost-effectiveness of esketamine relative to ECT remain under discussion. Further research is needed to clarify esketamine’s role in TRD treatment and to establish the best approach for long-term management.

**Disclosure of Interest:**

None Declared

